# Dr. Yeats, on the Discoveries of Mayow

**Published:** 1799-06

**Authors:** G. D. Yeats

**Affiliations:** Bedford


### *To the Editors of the Medical and Phyfical Journal.*

GENTLEMEN,

I SHALL feel obliged to you to infert, in your next number, the following obfervations on MAYOW'S DISCOVERIES, in anfwer to *Dr. Lubbock*, on the fame fubject.

I am fully convinced that the writings of Mayow fpread with confiderable rapidity throughout Europe, as can be eafily proved from Morhof and others. Notwithftanding, however, this diffufion of his doctrines, they do not appear to have been generally received in the full and ftrict fenfe in which Mayow wifhed them to be underftood: almoft all the writers who mention his doctrine confounded his fire-air particles, with the particles of nitre. From this circumftance it is, as I have faid in the introduction, " that many by not underftanding him, rejected his opinions, and others attributed to him doctrines which he never held." Among thefe we may particularly mention Schelhamerus, who exclaims with a degree of farcaftic envy. *Quæ fententia*, (the abforption of nitrous particles by the lungs) *ut eft novitatum amans hoc feculum*, ftatim ab omnibus vel tantum non omnibus Anglis, Batavis, Gallis, Germanis, avide arrepta eft. De nitro, p. 100. This fubject is fully explained in obfervations on modern difcoveries. p. 240. Two of the authors alfo quoted by Dr. Lubbock, viz. Dr. Connor and Mr. Tabor, have made no difcrimination between particles of nitre, and fire-air particles; the others may have likewife fallen into the fame error, but this I cannot affert, not having feen their works, and the paffages are not detailed.

Although I do not believe that Mayow's doctrines were favourably received, immediately upon their publication, yet I am far from afferting, with Dr. Beddoes, that his name was never echoed by popular applaufe; and, at the end of the 14th Sect. of my work, I have faid, that I was happy, from the inveftigation gone through, in being able to contradict this too hafty conclufion; for his opinions had been adopted and commented upon by

Wolfer-

Wolferſtan, Baglivi, Morhof, Collins, Verhayen, Hales, Drake, and Haller, and it affords me ſenſible ſatisfaction, to add the names mentioned by Dr. Lubbock.

During Mayow's life, however, not much notice ſeems to have been taken of his writings. In no work which I have conſulted, publiſhed during his life, do I find his name mentioned, or a diſtant hint of his doctrine: and what appears to me ſurpriſing is, that in a theſis which Thruſton read for his degree at Cambridge, in 1664, but which was not publiſhed for ſeveral years after, he makes no mention of Mayow, notwithſtanding he entertains ideas very ſimilar to Mayow's concerning reſpiration. Thruſton's opinion, too, had been freely canvaſſed by ſome, as he himſelf tells us, "quod theoriæ meæ, a nonnullis publicè ſed ſuppreſſo meo nomine, impugnatæ." Lower's 'Tractatus de Corde' was publiſhed during Mayow's life, and the fourth edition, twelve years after Mayow had given to the world his 'Experiments and Obſervations on Reſpiration;' yet not a word concerning Mayow is to be found, notwithſtanding Lower was one of the firſt who obſerved the change of colour which the blood acquires in the lungs, and which was ſo beautifully illuſtrated and confirmed by the ingenious experiments of Mayow, vide pp. 105 and 124 of 'Obſervations on Modern Diſcoveries.' The Reviewer in the 'Phil. Tranſact.' did not once ſmile upon his labours; Jocker, in his 'Lexicon,' aſſerts he was not noticed during his life; and Wood, in his 'Athenæ Oxon.' gives but a very ſcanty and ſuperficial account of him, which would not have been the caſe, I think, had his works been earlier attended to. He died in 1679, and I do not find his name mentioned till the following year, twelve years after the publication of his Treatiſe on Reſpiration; a ſhort period, it will be ſaid, but from which we may, nevertheleſs, in ſome degree, infer a neglect of his opinions.

The philoſophy of Mayow was publiſhed in an age remarkable for its production of great literary characters. Cotemporary with him were Boyle, Newton, Lower, Willis, and innumerable others, whoſe labours have conſiderably improved the ſciences: Harvey had but lately diſcovered the circulation; the philoſophic world was engaged in examining the Newtonian and Carteſian doctrines; and the theory of Stahl was juſt adopted by chemiſts, and was in the full career of its glory. Germany was now famous, all over Europe, for its production of able chemiſts; and men were much attached to the chemical labours of that country, which had given birth to Becker, to Van Helmont, and to Stahl. It was impoſſible for a man who died at the early age of thirty-two, however true his theory, or juſt his ſyſtem, to oppoſe with ſucceſs ſuch accumulated obſtacles. No champion would be found hardy enough to ſtem the tide of popular opinion, with theories and experiments not his own; and it is not likely, that many would risk their literary reputation in defence of doctrines, which appeared to meet with general disapprobation. Thus it was that the opinions of Mayow did not attract notice till many years after his death, when his works were read and admired by some of the first philosophers of the age. His opinions, however, were not generally understood, so as to form a system; the public mind was not ripe for improvement, and thus it was that his theories were lost in the great mass of discordant doctrines.

I have certainly said, that Munday does not mention Mayow. The edition I used may be different from Dr. Lubbock's; at any rate, in addition to my want of leisure at the time, I found so much jargon, that it was with difficulty I got through as much as I did. I have been informed that there is a family now living in Cornwall of the same name with Mayow; and, according to Wood, he was descended from a family living at Bree, in the same county.

I shall always hold in veneration the extraordinary abilities of this great genius, the Newton (as Dr. Lubbock very deservedly styles him) of chemical science; and I feel happy in being able here to repeat, from further testimonies, what I have elsewhere observed, that "the name of this great man is now restored to the annals of science; his genius is acknowledged, his abilities admired, and his opinions confirmed. The unequivocal concurrence of respectable testimonies has added lustre to his literary fame; and may the laurel of merited reputation continually flourish, unfaded, which the fatigues of discovery have wreathed about his brow!"

I am, Gentlemen,

Your's, &c.

*Bedford, May* 10, 1799. G. D. YEATS.

